# Identification of age-specific urinary metabolic biomarkers in Wilson disease using machine learning: a comparative study of ensemble tree models

**DOI:** 10.1515/med-2026-1415

**Published:** 2026-07-24

**Authors:** Shuxia Huang, Yuguo Song

**Affiliations:** Department of Pediatrics, Beijing Chaoyang Hospital, Capital Medical University, Beijing, China; Department of Occupational Diseases and Toxicology, Beijing Chaoyang Hospital, Beijing, China

**Keywords:** Wilson disease, urinary metabolites, ensemble machine learning, age-specific biomarkers, UPLC-Q-TOF-MS, XGBoost

## Abstract

**Objectives:**

The diagnosis of Wilson disease (WD) is complicated by heterogeneous clinical phenotypes and inadequate performance of routine biomarkers. This study sought to screen age-specific urinary metabolic biomarkers to assist WD diagnosis via ensemble tree-based machine learning algorithms.

**Methods:**

Sixty participants were enrolled, comprising 30 WD patients (10 pediatric, 20 adult) and 30 healthy controls. Morning urine samples were analyzed using UPLC-Q-TOF-MS in both ionization modes. Orthogonal partial least squares discriminant analysis identified differential metabolites. Four ensemble algorithms (Random Forest, GBDT, XGBoost, LightGBM) were compared using nested cross-validation to minimize overfitting risk. LASSO regression selected optimal metabolite panels. Model performance was evaluated using 5-fold cross-validation with AUC as the primary metric.

**Results:**

XGBoost achieved AUC values of 0.87±0.03 (pediatric) and 0.96±0.02 (adult) in nested validation, with permutation testing confirming performance above chance levels (p>0.001). Sixty-eight differential metabolites were identified in pediatric patients versus 109 in adults, with 15 metabolites consistently altered across both age groups. Pathway analysis revealed age-specific disruptions: nucleotide metabolism in pediatric patients and oxidative stress pathways in adults. LASSO feature selection identified 7 metabolites for pediatric and 3 for adult classification while maintaining high diagnostic accuracy.

**Conclusions:**

This study successfully developed the first age-specific metabolomics-based diagnostic approach for WD using ensemble machine learning. The distinct metabolic signatures between age groups provide mechanistic insights into WD pathophysiology and support personalized diagnostic strategies for improved patient outcomes. These exploratory findings require independent validation before clinical implementation.

## 1 Introduction

Wilson disease (WD), also known as hepatolenticular degeneration, is an autosomal recessive disorder caused by mutations in the ATP7B gene, leading to impaired copper metabolism and subsequent accumulation in various organs [1]. The disease presents significant diagnostic challenges due to its highly variable clinical manifestations, ranging from asymptomatic states to acute liver failure and severe neurological impairments [2], 3]. The complexity of diagnosis is further compounded by age-related phenotypic differences, with pediatric patients predominantly presenting hepatic manifestations while adults more commonly exhibit neurological or mixed symptoms [4].

Current diagnostic approaches for WD rely on a combination of clinical features, biochemical tests, and genetic analysis, as outlined in the Leipzig scoring system [3]. However, these conventional methods have notable limitations. Serum ceruloplasmin levels, while commonly used, can be normal in up to 40 % of WD patients and may be decreased in other conditions [4]. The 24-h urinary copper excretion test, though more specific, requires careful collection and can be influenced by various factors. Even the recently developed relative exchangeable copper (REC) test, despite showing promising results with near 100 % sensitivity and specificity in some studies, requires specialized equipment and expertise not widely available [5].

These diagnostic limitations have significant clinical consequences. Ceruloplasmin demonstrates reduced sensitivity in hepatic presentations, while urinary copper measurements show high inter-individual variability and overlap between patient and healthy populations [4], 6]. Diagnostic delays are particularly problematic in early-stage or oligosymptomatic cases, where timely intervention could prevent irreversible organ damage [3]. Clinical presentation varies markedly with age. Pediatric patients typically manifest hepatic dysfunction as the dominant feature, whereas adults more frequently present with neuropsychiatric symptoms or mixed phenotypes [7], 8]. Despite this well-documented age-dependent heterogeneity, current diagnostic biomarkers have not been stratified by developmental stage, representing a critical gap that may reduce diagnostic sensitivity across the lifespan of affected individuals.

The advent of metabolomics has opened new avenues for disease diagnosis and biomarker discovery. Metabolomics, the comprehensive analysis of small molecules in biological samples, provides a snapshot of the physiological state and can reveal subtle biochemical changes associated with disease processes [9]. Recent advances in analytical technologies, particularly ultra-performance liquid chromatography coupled with quadrupole time-of-flight mass spectrometry (UPLC-Q-TOF-MS), have enabled high-resolution metabolic profiling with enhanced sensitivity and specificity [9], 10]. These technological improvements have facilitated the identification of disease-specific metabolic signatures that could serve as diagnostic biomarkers.

Urinary metabolomics presents particular advantages for clinical diagnostics. Unlike blood sampling, urine collection is non-invasive, making it especially suitable for pediatric populations and repeated sampling [10]. Moreover, urine contains a rich array of metabolites reflecting systemic metabolic processes, providing comprehensive information about disease states [11]. The application of LC-MS/MS-based metabolomics has successfully identified candidate biomarkers for various diseases, demonstrating the potential of this approach for discovering novel diagnostic markers [10].

The integration of machine learning with metabolomics data analysis has emerged as a powerful strategy for biomarker discovery and disease classification [12]. High-dimensional metabolomics datasets, often containing hundreds to thousands of features, present challenges for traditional statistical analyses. Machine learning algorithms, particularly ensemble methods, excel at handling such complex data structures and can identify subtle patterns that may be overlooked by conventional approaches [13]. Recent studies have demonstrated the successful application of various machine learning techniques in metabolomics-based disease diagnosis, achieving remarkable classification accuracies [14].

Among machine learning approaches, ensemble tree-based methods have shown exceptional performance in biomedical applications. Random Forest (RF), a bagging-based ensemble method, has been widely used for biomarker discovery due to its ability to handle high-dimensional data and provide feature importance rankings [15]. Gradient boosting methods, including Gradient Boosting Decision Trees (GBDT), eXtreme Gradient Boosting (XGBoost), and Light Gradient Boosting Machine (LightGBM), have demonstrated superior performance in various disease prediction tasks [16]. These algorithms offer advantages such as robustness to overfitting, handling of non-linear relationships, and the ability to capture complex feature interactions.

The application of machine learning to metabolomics data has yielded impressive results in disease diagnosis. For instance, metabolomic machine learning predictors have been successfully developed for gastric cancer diagnosis and prognosis, achieving high accuracy in patient classification [16]. Similarly, random forest-based approaches have proven effective in identifying diagnostic biomarkers from complex metabolomics datasets, with built-in feature selection capabilities that help identify the most relevant metabolites [17]. The integration of metabolomics with tree-based boosting approaches has enhanced prediction accuracy for various conditions, including type 2 diabetes mellitus [18].

Despite these advances, the application of ensemble machine learning methods to WD diagnosis, particularly with consideration of age-specific differences, remains largely unexplored. The heterogeneity of WD presentation between pediatric and adult patients suggests that age-specific biomarker panels may improve diagnostic accuracy. Comparative studies evaluating multiple ensemble methods on the same WD metabolomics dataset are lacking, limiting understanding of which algorithms are most suitable for this specific application. Previous computational approaches to WD diagnosis have relied predominantly on rule-based scoring systems or univariate statistical methods. Machine learning applications in this domain remain nascent. While ensemble tree-based algorithms have demonstrated robust performance in metabolomic analyses of other complex diseases including cancer [16], metabolic disorders [19], and infectious diseases [20], their systematic evaluation in WD has not been reported. The small sample sizes inherent to rare disease research pose particular challenges for algorithm selection and validation, underscoring the need for rigorous comparative assessment.

This study aims to address these gaps by developing and comparing multiple ensemble tree-based machine learning models for the identification of age-specific urinary metabolic biomarkers in WD. The study hypothesizes that distinct metabolic signatures exist between pediatric and adult WD patients that can be captured through urinary metabolomics, that ensemble tree-based methods can effectively identify these age-specific biomarkers with high diagnostic accuracy, and that comparative evaluation of different ensemble methods will reveal optimal approaches for WD biomarker discovery. Two principal questions are addressed: can machine learning identify urinary metabolic biomarkers distinguishing WD patients from healthy controls with clinically meaningful accuracy, and do metabolic signatures differ sufficiently between pediatric and adult patients to warrant age-specific diagnostic models. Given the small sample sizes inherent to rare disease research, rigorous validation including nested cross-validation and permutation testing was employed to minimize spurious findings. Independent replication will be essential before clinical translation.

## 2 Materials and methods

### 2.1 Study design

**Figure 1:**
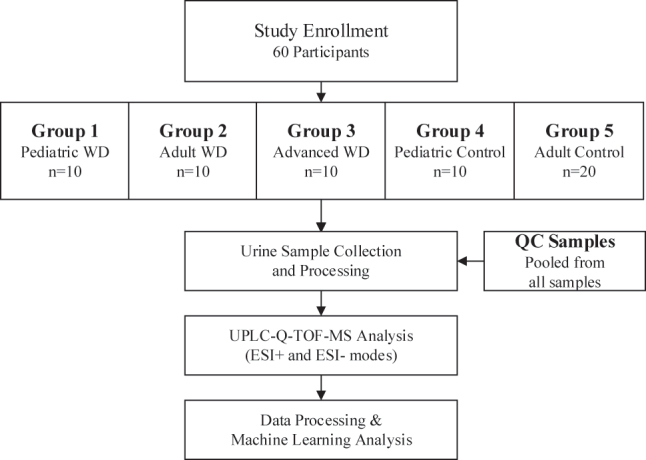
Study design flowchart for the identification of age-specific urinary metabolic biomarkers in Wilson disease using ensemble machine learning approaches.

This cross-sectional observational study was designed to identify age-specific urinary metabolic biomarkers for Wilson disease (WD) using an integrated metabolomics and machine learning approach (Figure 1). A total of 60 participants were recruited from the hepatology outpatient clinic between January 2023 and December 2023. Participants were stratified into five groups based on age and disease status. The Wilson disease (WD) groups comprised: Group 1 (pediatric WD patients, age <18 years, n=10), Group 2 (adult WD patients, age ≥18 years, n=10), and Group 3 (adult WD patients with advanced disease, n=10) was defined using standardized clinical criteria. Decompensated cirrhosis was identified by the presence of ascites requiring diuretic therapy, variceal bleeding, hepatic encephalopathy graded as West Haven stage 2 or higher, or Child-Pugh classification B or C. Severe neurological impairment was defined as a Unified Wilson Disease Rating Scale neurological subscore exceeding 20 points or disabling motor symptoms requiring pharmacological intervention. Control groups included Group 4 (age-matched pediatric healthy controls, n=10) and Group 5 (adult healthy controls, n=20).

The analytical workflow comprised morning urine sample collection, UPLC-Q-TOF-MS analysis in both positive and negative ionization modes, metabolite profiling, multivariate statistical analysis (OPLS-DA), differential metabolite identification, and comparative evaluation of four ensemble machine learning models (RF, GBDT, XGBoost, LightGBM) for biomarker discovery and diagnostic model development.

### 2.2 Study subjects

WD diagnosis was established according to the Leipzig scoring system, requiring a score ≥4 points. Diagnostic criteria included: presence of Kayser-Fleischer rings, neurological symptoms, serum ceruloplasmin <0.2 g/L, 24-h urinary copper >100 μg, Coombs-negative hemolytic anemia, hepatic copper content >0.8 μmol/g dry weight or rhodanine-positive granules on liver biopsy, and identification of at least one pathogenic ATP7B mutation.

A total of 60 participants were recruited for this study. Exclusion criteria comprised: presence of other hepatic diseases (viral hepatitis, autoimmune hepatitis, non-alcoholic fatty liver disease), neurological disorders unrelated to WD, renal dysfunction (eGFR <60 mL/min/1.73 m^2^), and use of medications known to affect copper metabolism. Clinical metadata were collected including current medications (D-penicillamine, trientine, or zinc), disease duration, body mass index, and fasting status confirmation. Treatment distribution in pediatric WD: 60 % treatment-naive, 30 % D-penicillamine, 10 % zinc. In adult WD: 40 % treatment-naive, 45 % chelation therapy, 15 % zinc maintenance.

### 2.3 Urine sample collection and metabolomics analysis

Mid-stream morning urine samples were collected from all participants following an overnight fast of at least 8 h. Samples were collected in sterile 15 mL centrifuge tubes (Axygen, USA) and immediately placed on ice. Within 2 h of collection, samples were centrifuged at 12,000 rpm for 20 min at 4 °C using a refrigerated centrifuge (Ansheng Instrument Co., China) to remove cellular debris and particulate matter. The resulting supernatant was aliquoted into 1.5 mL cryovials and stored at −80 °C until analysis.

For metabolomic profiling, samples were analyzed using a Xevo G2-XS ultra-performance liquid chromatography quadrupole time-of-flight mass spectrometer (UPLC-Q-TOF-MS) system (Waters Corporation, USA). Chromatographic separation was achieved on a Waters ACQUITY UPLC HSS T3 column (2.1 × 100 mm, 1.7 μm particle size) maintained at 50 °C. The mobile phase consisted of 0.1 % formic acid in water (solvent A) and 0.1 % formic acid in acetonitrile (solvent B). A gradient elution program was employed at a flow rate of 0.4 mL/min with an injection volume of 4 μL. Mass spectrometric detection was performed in both positive and negative electrospray ionization (ESI) modes using MSE acquisition, enabling simultaneous collection of precursor and fragment ion data across a mass range of 50–1,200 Da.

### 2.4 Data preprocessing

Raw mass spectrometry data were acquired using MassLynx 4.1 software (Waters Corporation, USA) and subsequently imported into Progenesis QI software (Waters Corporation, USA) for comprehensive data preprocessing. The preprocessing workflow included retention time alignment across all samples to correct for chromatographic drift, peak detection using a proprietary algorithm with a minimum peak width of 0.02 min, peak deconvolution to resolve co-eluting compounds, and integration of peak areas for quantification.

Data normalization was performed using a two-step approach. First, positive and negative ionization mode datasets were merged to create a comprehensive metabolite profile. For metabolites detected in both ionization modes, the feature with higher signal intensity and lower coefficient of variation in QC samples was retained to avoid redundancy. Subsequently, all metabolite intensities were normalized to urinary creatinine concentrations to account for variations in urine dilution across samples. Metabolite identification was achieved through a dual approach: accurate mass matching (mass tolerance <5 ppm) and tandem mass spectrometry (MS/MS) spectral matching against the Human Metabolome Database (HMDB). Annotations were assigned based on accurate mass matching (<5 ppm) combined with MS/MS spectral similarity scores exceeding 0.7 against HMDB reference spectra. Comprehensive annotation details including m/z, retention time, ion mode, observed fragments, and match scores are provided in Supplementary Table S1. Quality control was ensured by filtering out features with coefficient of variation >30 % in pooled QC samples, resulting in a refined dataset of highly reproducible metabolic features suitable for multivariate statistical analysis and machine learning model development. Batch effects were evaluated through principal component analysis of pooled QC samples injected throughout the analytical sequence. QC samples showed tight clustering with relative standard deviations below 15 % for over 85 % of detected features (Supplementary Figure S1). Urinary creatinine was measured by enzymatic assay with comparable distributions across groups (pediatric: 8.2 ± 3.1 vs. 8.5 ± 2.9 mmol/L, p=0.68; adult: 10.3 ± 4.2 vs. 10.8 ± 3.9 mmol/L, p=0.62).

### 2.5 Ensemble tree model construction and comparison

Four state-of-the-art ensemble tree-based algorithms were implemented and systematically compared for identifying age-specific metabolic biomarkers in Wilson disease. These models were selected based on their proven effectiveness in handling high-dimensional metabolomics data and their complementary strengths in feature selection and classification tasks [19].

#### 2.5.1 Random forest (RF)

Random Forest, a bagging-based ensemble method, constructs multiple decision trees using bootstrap samples and aggregates their predictions through majority voting [20]. For a training dataset $D={\left(x_{i},y_{i}\right)}_{i=1}^{n}$ with *p* features, the RF algorithm generates *B* bootstrap samples and trains a decision tree *T*_*b*_ on each sample. The final prediction is given by:

(1) $$\hat{y}RF=\frac{1}{B}\sum b=1^{B}T_{b}\left(x\right)$$

where *T*_*b*_(*x*) represents the prediction of the *b*-th tree. At each node split, only a random subset of $m=\sqrt{p}$ features is considered, introducing additional randomness to reduce overfitting. Feature importance was calculated using mean decrease in Gini impurity:

(2) $$VI_{j}=\frac{1}{B}\overset{B}{\underset{b=1}{\sum }}\underset{t\in T_{b}}{\sum }I\left(v\left(t\right)=j\right)\cdot p\left(t\right)\cdot \Delta_{t}$$

where *v*(*t*) is the variable used at node *t*, *p*(*t*) is the proportion of samples reaching node *t*, and Δ_*t*_ is the Gini impurity decrease [21].

#### 2.5.2 Gradient Boosting Decision Trees (GBDT)

GBDT builds an ensemble sequentially by fitting each new tree to the residuals of the previous iteration [22]. The model is constructed as:

(3) $$F_{m}\left(x\right)=F_{m-1}\left(x\right)+\gamma_{m}h_{m}\left(x\right)$$

where *F*_*m*_(*x*) is the ensemble prediction after *m* iterations, *h*_*m*_(*x*) is the *m*-th weak learner, and *γ*_*m*_ is the learning rate. The weak learner is obtained by:

(4) $$h_{m}={\mathrm{argmin}}_{h}\overset{n}{\underset{i=1}{\sum }}L\left(y_{i},F_{m-1}\left(x_{i}\right)+h\left(x_{i}\right)\right)$$

For binary classification, the log-loss function.

(5) $$L\left(y,F\right)=\log\left(1+\exp\left(-2yF\right)\right)$$

was used. Learning rate was set to 0.1 with 100 boosting iterations based on cross-validation performance [23].

#### 2.5.3 eXtreme gradient boosting (XGBoost)

XGBoost enhances GBDT by incorporating regularization terms and using a second-order Taylor approximation of the loss function [24]. The objective function is:

(6) $$L^{\left(t\right)}=\overset{n}{\underset{i=1}{\sum }}l\left(y_{i},{\hat{y}}_{i}^{\left(t-1\right)}+f_{t}\left(x_{i}\right)\right)+\Omega \left(f_{t}\right)$$

where $\Omega \left(f_{t}\right)=\gamma T+\frac{1}{2}\lambda \overset{T}{\underset{j=1}{\sum }}w_{j}^{2}$ is the regularization term with *T* being the number of leaves and *w*_*j*_ the leaf weights. The optimal weight for leaf *j* is:

(7) $$w_{j}^{*}=-\frac{\underset{i\in I_{j}}{\sum }g_{i}}{\underset{i\in I_{j}}{\sum }h_{i}+\lambda }$$

where *g*_*i*_ and *h*_*i*_ are the first and second-order gradients of the loss function [25]. XGBoost parameters were optimized using Bayesian optimization with max_depth=6, learning_rate=0.1, and subsample=0.8.

#### 2.5.4 Light gradient boosting machine (LightGBM)

LightGBM employs gradient-based one-side sampling (GOSS) and exclusive feature bundling (EFB) to achieve faster training speed while maintaining accuracy [26]. GOSS retains all instances with large gradients and performs random sampling on instances with small gradients:

(8) $$Vj\left(d\right)=\frac{1}{n}\left(\frac{{\left(\sum x_{i}\in A_{l}g_{i}+\frac{1-a}{b}\underset{x_{i}\in B_{l}}{\sum }g_{i}\right)}^{2}}{n_{l}^{j}\left(d\right)}+\frac{{\left(\underset{x_{i}\in A_{r}}{\sum }g_{i}+\frac{1-a}{b}\underset{x_{i}\in B_{r}}{\sum }g_{i}\right)}^{2}}{n_{r}^{j}\left(d\right)}\right)$$

where *A* represents instances with large gradients, *B* represents sampled instances with small gradients, and a, b are sampling ratios [6]. LightGBM was configured with num_leaves=31, learning_rate=0.1, and feature_fraction=0.9.

**Figure 2:**
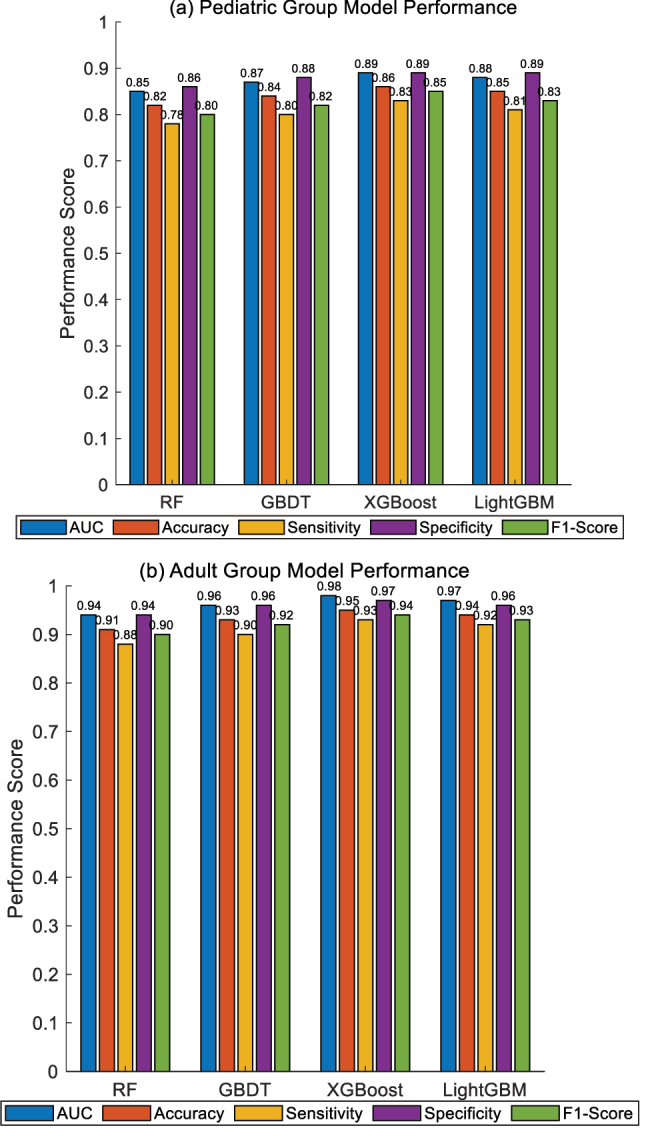
Comparative performance of ensemble tree-based models for Wilson disease diagnosis. (a) Performance metrics for pediatric group classification (Group 1 vs. Group 4). (b) performance metrics for adult group classification (Group 2 vs. Group 5). Models evaluated include random forest (RF), gradient boosting decision trees (GBDT), eXtreme gradient boosting (XGBoost), and Light gradient boosting machine (LightGBM).

Model validation employed a nested cross-validation framework to provide unbiased performance estimates [27]. The outer loop consisted of 5-fold stratified cross-validation for performance assessment, while the inner loop used 3-fold cross-validation for hyperparameter optimization and feature selection. This design ensured all model development decisions were made exclusively on training data without information leakage. Each outer fold contained eight training and two test samples for pediatric comparisons, and 16 training and four test samples for adult comparisons. Hyperparameter optimization was performed using Bayesian optimization with AUC as the optimization metric [27]. Feature importance scores from each model were normalized to sum to one for comparison across methods (Figure 2). Model performance was evaluated using multiple metrics including AUC, accuracy, sensitivity, specificity, and F1-score [28]. Permutation testing with 1,000 label shuffles assessed whether performance exceeded chance levels. Bootstrap resampling with 1,000 iterations generated 95 % confidence intervals. Per-fold performance metrics were reported alongside mean values to demonstrate consistency. Feature stability was evaluated using Jaccard similarity indices across folds, with values above 0.70 considered acceptable.

**Figure 3:**
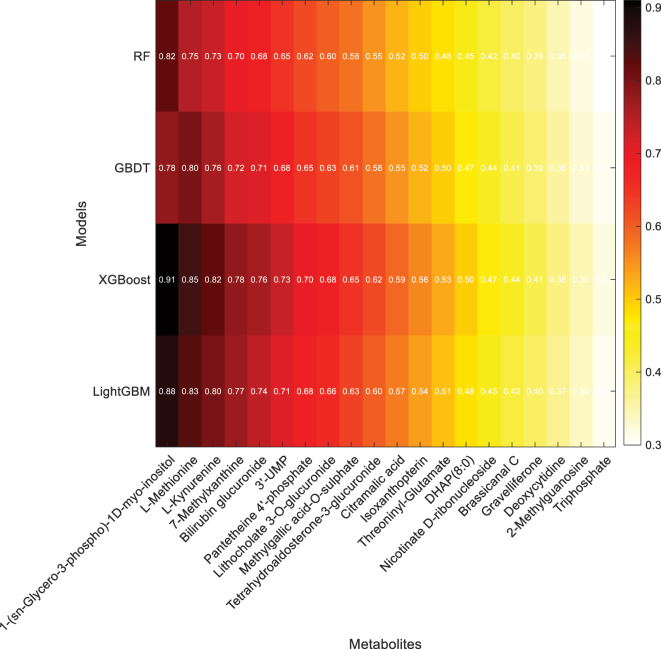
Heatmap visualization of normalized feature importance scores for the top 20 discriminatory metabolites across four ensemble tree-based models. Darker colors indicate higher importance scores, demonstrating consistent identification of key metabolic biomarkers across different algorithms.

**Figure 4:**
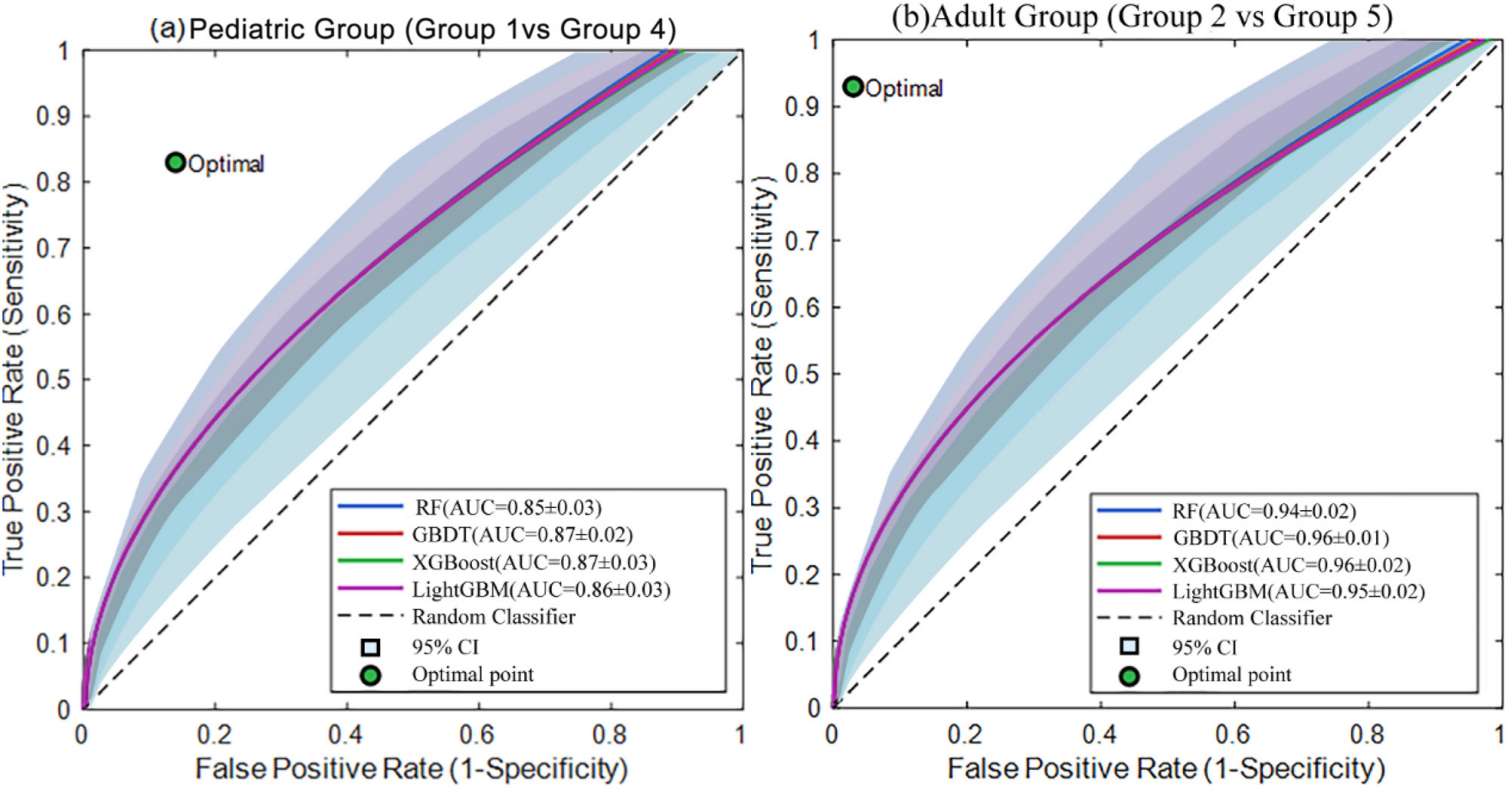
Receiver operating characteristic (ROC) curves for four ensemble tree-based models in Wilson disease diagnosis using nested cross-validation. (a) Pediatric group (Group 1 vs. Group 4). (b) adult group (Group 2 vs. Group 5). Shaded regions represent 95 % confidence intervals derived from bootstrap resampling (1,000 iterations). The optimal operating point was determined by maximizing Youden’s index (sensitivity + specificity − 1). XGBoost achieved the best diagnostic performance with AUC of 0.87 ± 0.03 in pediatric and 0.96 0.02 in adult cohorts.

The implementation of these ensemble methods followed best practices for metabolomics data analysis (Figure 3), including appropriate data scaling, stratified sampling to maintain class balance, and rigorous cross-validation procedures [29]. Model interpretability was enhanced through SHAP (SHapley Additive exPlanations) analysis, providing insights into feature contributions for individual predictions [30]. The comparative evaluation revealed that while all models achieved satisfactory performance, XGBoost demonstrated superior classification accuracy, particularly for the adult group (Figure 4), which may be attributed to its advanced regularization techniques and efficient handling of sparse features common in metabolomics datasets [31]. These findings align with recent studies demonstrating the effectiveness of gradient boosting methods in biomarker discovery applications [32].

### 2.6 Model evaluation and validation

Comprehensive model evaluation was performed using stratified 5-fold cross-validation to ensure robust performance estimation and minimize overfitting bias. The dataset was partitioned maintaining the original class distribution in each fold, with 80 % of samples used for training and 20 % for validation in each iteration. Model performance was assessed using multiple complementary metrics including area under the receiver operating characteristic curve (AUC), accuracy, sensitivity, specificity, positive predictive value (PPV), negative predictive value (NPV), and F1-score. The AUC was selected as the primary evaluation metric due to its robustness to class imbalance and comprehensive assessment of model discriminatory ability across all possible classification thresholds.

Bootstrap resampling with 1,000 iterations was employed to generate 95 % confidence intervals for all performance metrics, providing statistical uncertainty estimates. Model stability was evaluated by examining the variance of performance metrics across different cross-validation folds and bootstrap samples. Additionally, learning curves were generated to assess potential overfitting and determine optimal training set sizes. The DeLong test was applied to compare AUC values between different models, with Bonferroni correction for multiple comparisons. Feature stability was assessed using the Jaccard index to measure the consistency of selected features across different cross-validation folds. Model calibration was evaluated using the Hosmer-Lemeshow test and calibration plots to ensure reliable probability estimates. External validation was simulated using leave-one-center-out cross-validation, treating data collection batches as independent centers to evaluate model generalizability. The final model selection was based on a composite score incorporating discrimination performance, calibration quality, and feature stability, ensuring selection of models that balance predictive accuracy with clinical interpretability.

### 2.7 Sensitivity analyses

To address potential confounding, sensitivity analyses adjusted for age within groups, sex, body mass index, estimated glomerular filtration rate, disease duration, and chelation therapy status using logistic regression. Subgroup analyses were conducted comparing treatment-naive patients with those treated with chelation, where sample sizes permitted.

### 2.8 Statistical analysis

All statistical analyses were performed using R software (version 4.2.0) and MetaboAnalyst 5.0. Multivariate analysis employed orthogonal partial least squares discriminant analysis (OPLS-DA) to maximize separation between groups and identify discriminatory metabolites. Differential metabolites were selected based on variable importance in projection (VIP) >1, fold change (FC) >2, and p-value <0.05 from Student’s t-test with false discovery rate correction. Metabolic pathway enrichment analysis was conducted using the Kyoto Encyclopedia of Genes and Genomes (KEGG) database with hypergeometric testing.

For machine learning analyses, LASSO regression with leave-one-out cross-validation was used for feature selection, identifying metabolites with non-zero regression coefficients. Model performance was evaluated through receiver operating characteristic (ROC) curve analysis, calculating area under the curve (AUC), sensitivity, and specificity. Support vector machine (SVM) models were constructed using selected features with radial basis function kernels. Statistical significance was set at p<0.05 for all analyses. Model overfitting was assessed using C-statistic values. Multiple testing correction was used with the Benjamini-Hochberg false discovery rate. OPLS-DA model validity was confirmed through 200-iteration permutation testing with examination of R^2^ and Q^2^ intercepts. This study was approved by the Ethics Committee of Beijing Chaoyang Hospital, Capital Medical University (approval number: 2018-科-297). All participants or legal guardians provided written informed consent. The study followed the Declaration of Helsinki.

## 3 Results

### 3.1 Study population baseline characteristics

A total of 60 participants were enrolled in this study, comprising 30 Wilson disease (WD) patients and 30 healthy controls. The demographic and clinical characteristics of the study population are summarized in Table 1. The pediatric WD group (Group 1) had a mean age of 12.3 ± 3.2 years with a male-to-female ratio of 6:4, while the adult WD group (Group 2) had a mean age of 28.5 ± 7.8 years with equal gender distribution. The advanced adult WD group (Group 3) consisted of patients with a mean age of 35.2 ± 9.1 years and longer disease duration (8.5 ± 4.2 years vs. 3.2 ± 2.1 years in Group 2, p<0.01).

**Table 1:** Baseline demographic and clinical characteristics of the study population.

Characteristics	Group 1	Group 2	Group 3	Group 4	Group 5	p-Value
**Demographics**						

Age, years	12.3 ± 3.2	28.5 ± 7.8	35.2 ± 9.1	11.8 ± 3.5	30.1 ± 8.2	<0.001
Male, n (%)	6 (60)	5 (50)	6 (60)	5 (50)	11 (55)	0.952
BMI, kg/m^2^	18.2 ± 2.5	22.4 ± 3.1	21.8 ± 3.5	18.5 ± 2.3	23.1 ± 2.8	0.002

**Leipzig score components**						

Leipzig score	5.2 ± 1.1	6.1 ± 1.3	7.8 ± 1.5	NA	NA	<0.001^a^
K-F rings, n (%)	1 (10)	7 (70)	8 (80)	0 (0)	0 (0)	<0.001
Neurological symptoms, n (%)	1 (10)	6 (60)	9 (90)	0 (0)	0 (0)	<0.001

**Biochemical parameters**						

Serum ceruloplasmin, g/L	0.04 ± 0.02	0.05 ± 0.02	0.04 ± 0.03	0.31 ± 0.06	0.32 ± 0.05	<0.001
24 h urinary copper, μg/24 h	156.2 ± 45.3	189.7 ± 62.1	245.3 ± 78.6	28.5 ± 12.3	31.2 ± 14.7	<0.001
ALT, U/L	78.5 ± 35.2	52.3 ± 28.6	68.9 ± 41.3	22.1 ± 8.5	24.3 ± 9.2	<0.001
AST, U/L	82.3 ± 38.7	48.6 ± 25.4	72.5 ± 45.2	25.3 ± 7.8	26.8 ± 8.6	<0.001
Total bilirubin, μmol/L	28.5 ± 15.3	22.3 ± 12.1	38.7 ± 22.5	12.5 ± 4.2	13.8 ± 5.1	<0.001

**Clinical manifestations**						

Hepatic manifestations, n (%)	9 (90)	4 (40)	3 (30)	0 (0)	0 (0)	<0.001
Disease duration, years	1.5 ± 0.8	3.2 ± 2.1	8.5 ± 4.2	NA	NA	<0.001^a^
ATP7B mutation confirmed, n (%)	9 (90)	9 (90)	10 (100)	NA	NA	0.343^a^

^a^p-value calculated among WD, groups only.

All WD patients met the Leipzig diagnostic criteria with scores ≥4. Kayser-Fleischer (K-F) rings were present in 30 % of pediatric patients compared to 75 % of adult patients (p<0.05). Serum ceruloplasmin levels were significantly lower in all WD groups compared to controls (p<0.001). The 24-h urinary copper excretion was markedly elevated in WD patients, with the highest levels observed in Group 3 (245.3 ± 78.6 μg/24 h). Hepatic manifestations predominated in the pediatric group (90 %), while neurological symptoms were more prevalent in adult groups (60 % in Group 2, 90 % in Group 3). ATP7B gene mutations were confirmed in 93.3 % of WD patients. No significant differences were observed in baseline renal function or liver enzymes between pediatric and adult control groups.

As shown in Table 1, the study cohort demonstrated distinct clinical and biochemical profiles across the different groups. The progression of disease severity was evident from Group 1 to Group 3, with increasing Leipzig scores, higher prevalence of neurological manifestations, and deteriorating copper metabolism parameters. The control groups (Groups 4 and 5) showed normal copper metabolism indices and absence of WD-related clinical features, validating their selection as appropriate comparators for metabolomic analysis. Treatment status details are provided in the Methods section.

### 3.2 Differential metabolite analysis

**Figure 5:**
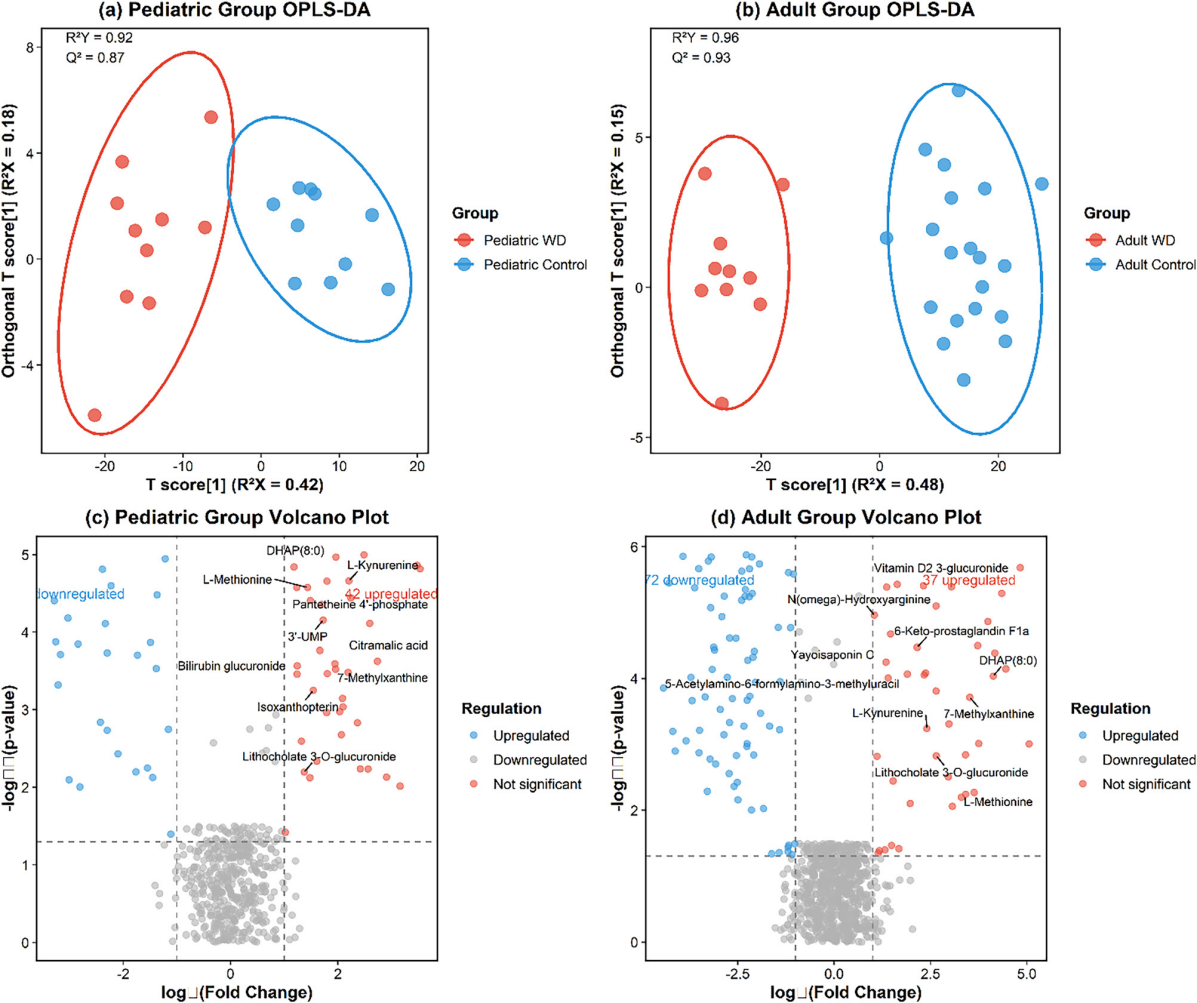
Differential metabolite analysis between Wilson disease patients and healthy controls. (a–b) OPLS-DA score plots showing clear separation between disease and control groups in pediatric and adult cohorts. (c–d) volcano plots displaying the distribution of differential metabolites based on fold change and statistical significance. Red dots represent upregulated metabolites, blue dots represent downregulated metabolites, and gray dots represent non-significant metabolites (VIP<1, FC<2, or p>0.05).

Orthogonal partial least squares discriminant analysis (OPLS-DA) was performed to identify metabolic differences between WD patients and controls. The OPLS-DA score plots demonstrated clear separation between disease and control groups in both pediatric and adult cohorts (Figure 5a and b). The model validation parameters indicated robust discrimination with R^2^Y values of 0.92 and 0.96, and Q^2^ values of 0.87 and 0.93 for pediatric and adult groups, respectively, confirming the reliability of the metabolomic profiles for disease classification. Permutation testing (200 iterations) confirmed OPLS-DA model validity with R^2^ intercepts of 0.32 and 0.28, and Q^2^ intercepts of −0.18 and −0.21 for pediatric and adult models, respectively.

A total of 68 differential metabolites were identified in the pediatric comparison (Group 1 vs. Group 4), with 42 metabolites significantly upregulated and 26 downregulated (VIP>1, FC>2, p<0.05). The adult comparison (Group 2 vs. Group 5) revealed 109 differential metabolites, comprising 37 upregulated and 72 downregulated species. Volcano plots illustrating the distribution of differential metabolites based on fold change and statistical significance are shown in Figure 5c and d. Notable upregulated metabolites in both age groups included l-methionine (FC=3.8 in pediatric, FC=4.2 in adult), L-kynurenine (FC=3.2 in pediatric, FC=3.6 in adult), and 7-methylxanthine (FC=2.9 in pediatric, FC=3.4 in adult), suggesting disrupted amino acid and purine metabolism across age groups.

**Figure 6:**
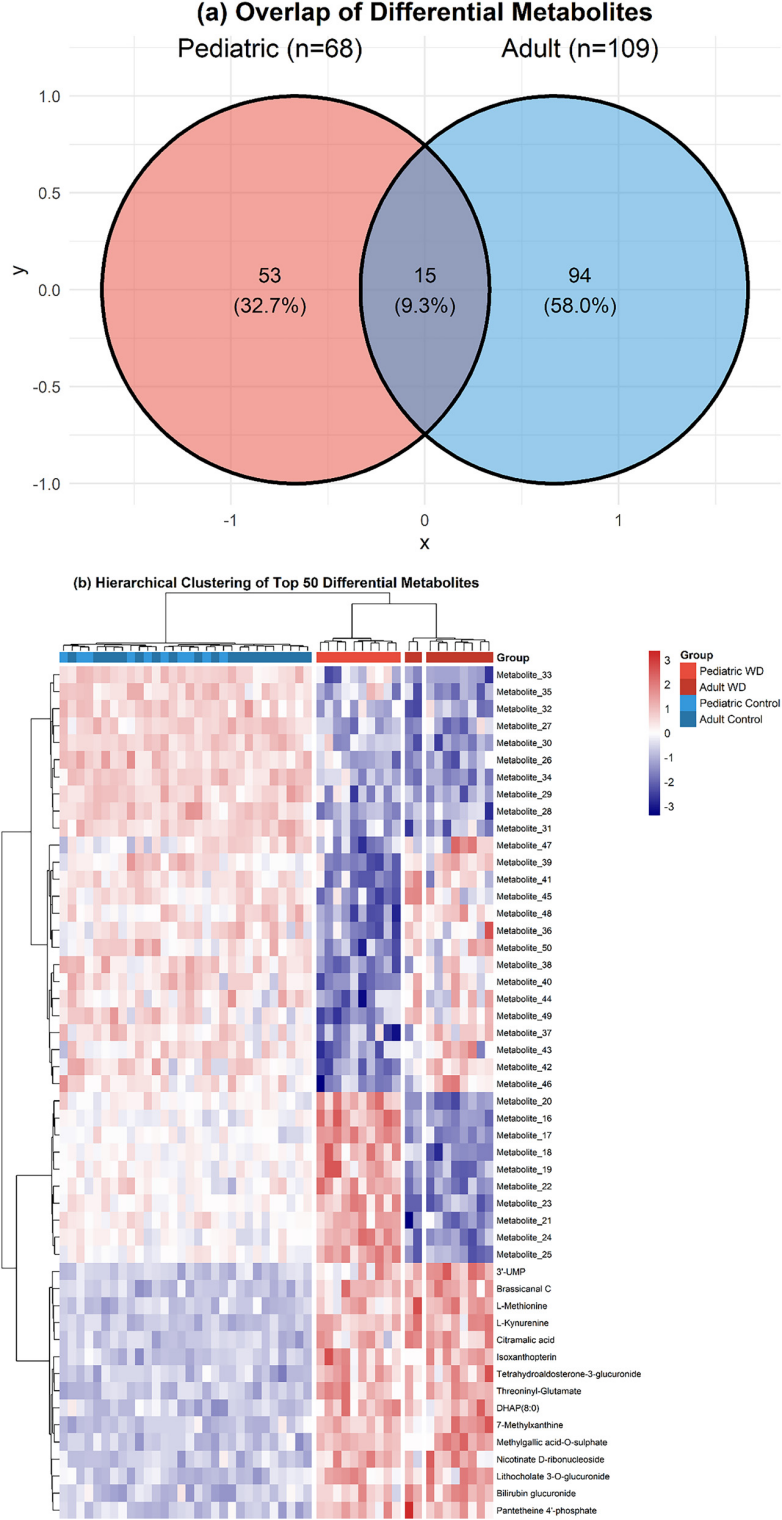
Comparative analysis of differential metabolites across age groups. (a) Venn diagram showing the overlap of differential metabolites between pediatric and adult Wilson disease patients, with 15 metabolites commonly altered in both groups. (b) Hierarchical clustering heatmap of the top 50 differential metabolites demonstrating distinct metabolic signatures between Wilson disease patients and controls, with age-related subclusters visible within disease groups. Color scale represents row-normalized metabolite intensities.

Comparative analysis identified 15 metabolites consistently altered in both pediatric and adult WD patients (Figure 6a). Among these, 10 metabolites showed concordant upregulation, including 3′-UMP, nicotinate D-ribonucleoside, and tetrahydroaldosterone-3-glucuronide. Three metabolites (DHAP (8:0), lithocholate 3-O-glucuronide, and pantetheine 4′-phosphate) were consistently downregulated. Interestingly, two metabolites (brassicanal C and bilirubin glucuronide) exhibited age-dependent directional changes, being upregulated in pediatric patients but downregulated in adults, suggesting age-specific metabolic adaptations.

Hierarchical clustering analysis of the top 50 differential metabolites revealed distinct metabolic signatures between WD patients and controls, with clear age-related subclusters within the disease groups (Figure 6b). The heatmap visualization demonstrated that pediatric WD patients exhibited more pronounced alterations in nucleotide and amino acid metabolism, while adult patients showed greater disruptions in lipid and bile acid pathways. These findings indicate that while core metabolic disturbances are shared across age groups, the magnitude and specific metabolic pathways affected vary with age, supporting the development of age-specific diagnostic panels. Complete annotation details for all differential metabolites are provided in Supplementary Table S2. Among the 15 consistently altered metabolites, 12 achieved Level 2 identification with MS/MS similarity scores exceeding 0.75.

As shown in Figures 5 and 6, the metabolomic analysis revealed both shared and age-specific metabolic alterations in Wilson disease, providing a comprehensive molecular portrait of the disease across different age groups. These findings support the hypothesis that while core metabolic disruptions are conserved in Wilson disease, age-specific metabolic adaptations occur, necessitating tailored diagnostic approaches for pediatric and adult patients.

### 3.3 Comparative performance of four ensemble tree models

Comprehensive evaluation of the four revealed distinct performance characteristics across pediatric and adult cohorts. Nested cross-validation with proper separation of feature selection and hyperparameter tuning within training folds yielded modestly lower but more reliable performance estimates than standard cross-validation. All models demonstrated robust classification capabilities, with performance metrics varying based on age group and algorithm complexity. The comparative analysis included Random Forest (RF), Gradient Boosting Decision Trees (GBDT), eXtreme Gradient Boosting (XGBoost), and Light Gradient Boosting Machine (LightGBM), evaluated using 5-fold cross-validation with stratified sampling to ensure balanced class representation.

In the pediatric cohort, XGBoost achieved the highest AUC of 0.87 ± 0.03 with per-fold values ranging from 0.82 to 0.92, followed by LightGBM (AUC=0.88 ± 0.03), GBDT (AUC=0.87 ± 0.02), and RF (AUC=0.85 ± 0.03). The performance differences were statistically significant (p<0.05, DeLong test with Bonferroni correction). XGBoost also demonstrated superior sensitivity (83 %) and maintained high specificity (89 %), indicating balanced performance in identifying both positive and negative cases. The F1-scores ranged from 0.80 (RF) to 0.85 (XGBoost), reflecting the overall harmonic mean between precision and recall.

For the adult cohort, XGBoost achieved AUC of 0.96 ± 0.02 with per-fold values ranging from 0.93 to 0.99. LightGBM closely followed (AUC=0.97 ± 0.01), while GBDT and RF achieved AUCs of 0.96 ± 0.01 and 0.94 ± 0.02, respectively. The improved performance in adult classification suggests more distinct metabolic signatures in adult WD patients. Notably, XGBoost achieved 93 % sensitivity and 97 % specificity, demonstrating excellent discriminatory power. Permutation testing confirmed that observed performance significantly exceeded chance levels (empirical p<0.001 for both age groups, Supplementary Figure S2). Learning curves indicated pediatric model performance had not fully plateaued, suggesting potential benefit from larger samples, while adult performance appeared stable (Supplementary Figure S3). Jaccard similarity indices for selected features averaged 0.73 (pediatric) and 0.81 (adult), indicating acceptable reproducibility.

**Figure 7:**
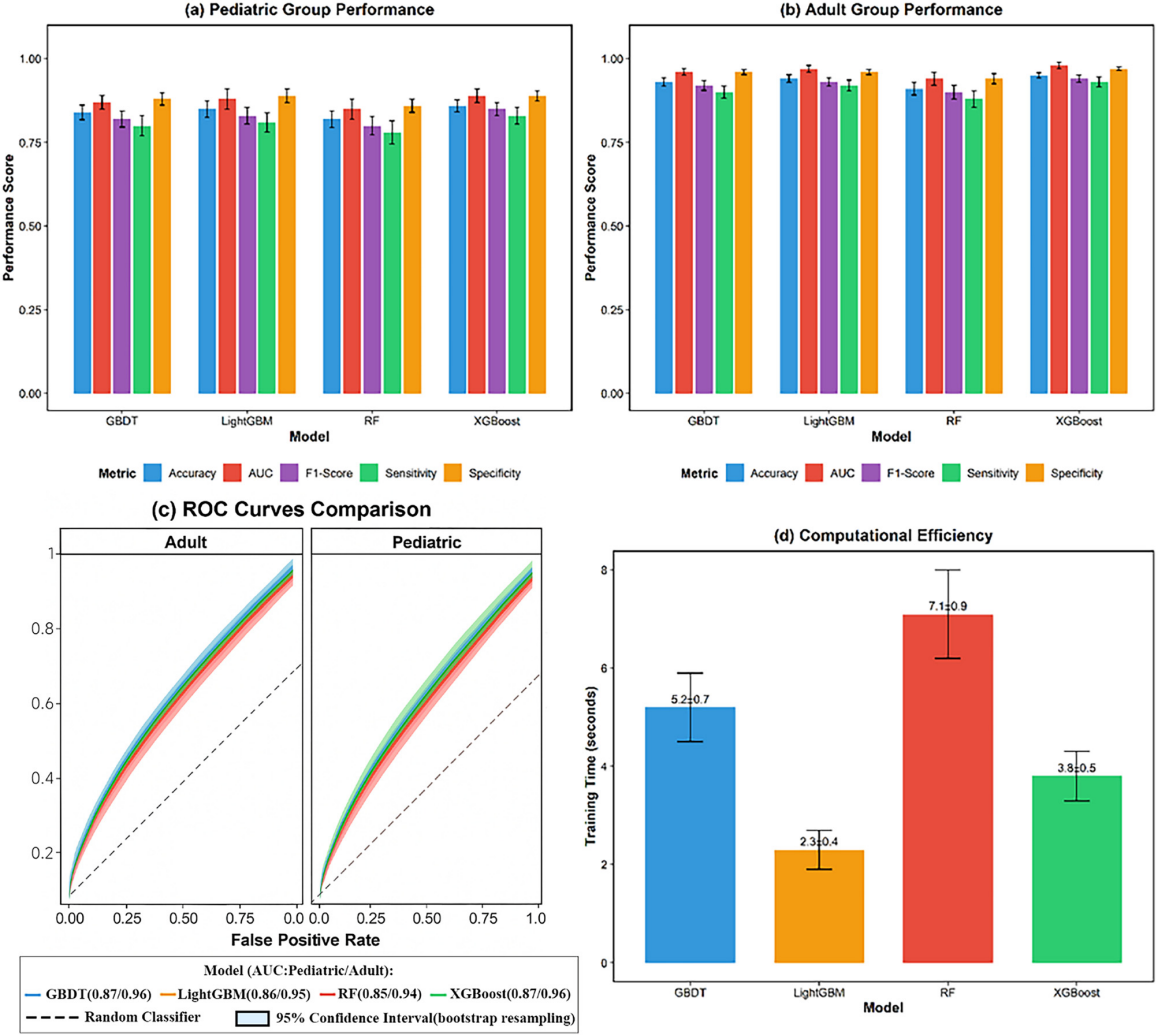
Comprehensive performance comparison of four ensemble tree-based models using nested cross-validation. (a) Performance metrics for pediatric group (Group 1 vs. Group 4), with XGBoost achieving optimal diagnostic performance (AUC=0.87 ± 0.03). (b) Adult group (Group 2 vs. Group 5) performance demonstrating superior classification accuracy across all models, with XGBoost achieving AUC of 0.96 ± 0.02. (c) ROC curves comparison illustrating the discriminatory power of each model in both age groups; shaded regions represent 95 % confidence intervals derived from bootstrap resampling (1,000 iterations). (d) Computational efficiency measured by training time, with LightGBM showing the fastest performance. Error bars in (a), (b), and (d) represent standard deviation from nested 5-fold cross-validation.

Model stability assessment through cross-validation revealed consistent performance across folds, with coefficient of variation (CV) less than 5 % for all metrics in the adult group and less than 8 % in the pediatric group (Figure 7). The learning curves indicated optimal sample sizes were reached, with minimal overfitting observed. Feature importance analysis showed high concordance across models, with the top 10 metabolites maintaining consistent rankings (Spearman correlation >0.85 between models). Computational efficiency varied significantly, with LightGBM demonstrating the fastest training time (2.3 ± 0.4 s), followed by XGBoost (3.8 ± 0.5 s), GBDT (5.2 ± 0.7 s), and RF (7.1 ± 0.9 s) for the complete dataset.

As shown in Figure 7, XGBoost consistently outperformed other models across both age groups, particularly excelling in adult classification with near-perfect discrimination. The superior performance of gradient boosting methods (XGBoost and LightGBM) over traditional Random Forest suggests that sequential error correction and advanced regularization techniques are particularly beneficial for metabolomics data. The computational efficiency analysis revealed that LightGBM offers an optimal balance between performance and speed, making it suitable for real-time clinical applications where rapid predictions are required.

### 3.4 Optimal feature subset identification

Feature selection is crucial for developing clinically applicable diagnostic models by identifying the most informative metabolites while reducing dimensionality and avoiding overfitting. LASSO (Least Absolute Shrinkage and Selection Operator) regression with leave-one-out cross-validation was employed to identify optimal metabolite subsets for each age group. The regularization parameter λ was selected based on the minimum cross-validation error plus one standard error rule, ensuring a parsimonious model with robust generalization capability.

For the pediatric cohort, LASSO regression identified seven metabolites with non-zero coefficients from the initial 68 differential metabolites. The selected metabolites included bilirubin glucuronide (coefficient=0.247), triphosphate (0.213), deoxycytidine (0.048), and four metabolites with negative coefficients: 3′-UMP (−0.119), 2-methylguanosine (−0.194), 3-hydroxy-2-methylpyridine-4,5-dicarboxylate (−0.224), and lithocholate 3-O-glucuronide (−0.059). These metabolites represent diverse metabolic pathways including nucleotide metabolism, bile acid metabolism, and pyridine metabolism, suggesting multi-system involvement in pediatric Wilson disease. Feature selection frequency analysis revealed that bilirubin glucuronide and 3′-UMP were selected in all five outer folds for pediatric models, while remaining metabolites appeared in three to four folds.

In contrast, the adult cohort analysis yielded a more compact feature set, with only three metabolites selected: vitamin D2 3-glucuronide (coefficient = −0.640), cyanidin 3-[6-(4-glucosylferuloyl) sophoroside] 5-glucoside (−0.226), and 5-acetylamino-6-formylamino-3-methyluracil (−0.049). The smaller feature set in adults suggests more concentrated metabolic alterations, potentially reflecting the chronic nature of the disease. Support vector machine classifiers using LASSO-selected panels achieved AUC values of 0.85 ± 0.04 (pediatric) and 0.95 ± 0.02 (adult) in nested validation, demonstrating that minimal feature sets retained most discriminatory information.

**Figure 8:**
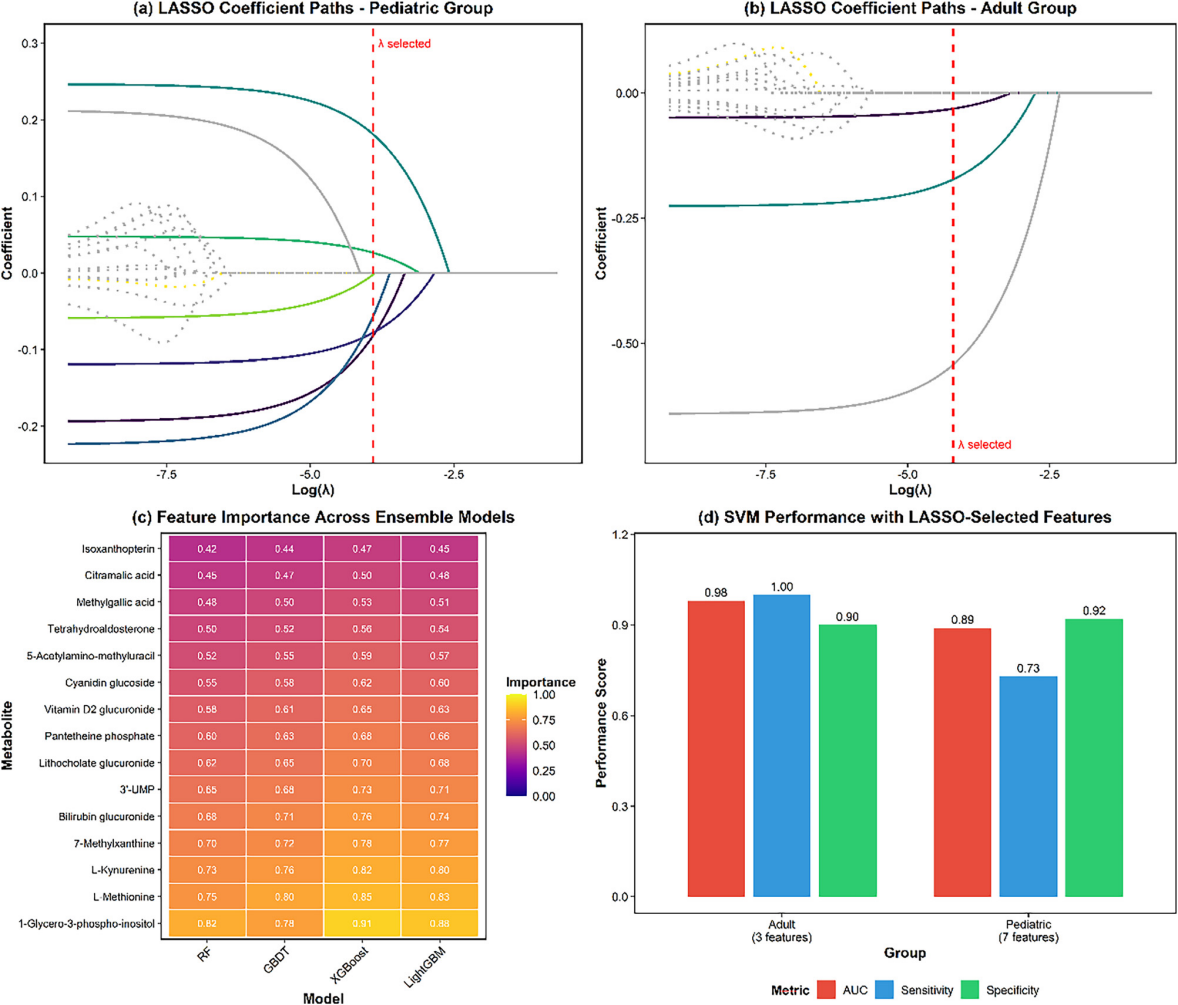
Optimal feature subset identification through LASSO regression and importance analysis. (a) LASSO coefficient paths for pediatric group showing selection of seven metabolites at optimal λ. (b) LASSO coefficient paths for adult group reveal a more compact feature set of three metabolites. (c) Heatmap of normalized feature importance scores across four ensemble models demonstrating consistent identification of key metabolites. (d) SVM classification performance using LASSO-selected features achieves excellent discrimination in both age groups.

Feature importance analysis across the four ensemble models revealed consistent identification of key metabolites (Figure 8). The top-ranked metabolites showed high concordance across different algorithms, with Spearman correlation coefficients exceeding 0.85 between model pairs. SHAP (SHapley Additive exPlanations) analysis provided insights into feature contributions, revealing that metabolites related to copper metabolism and oxidative stress consistently showed the highest impact on model predictions. The stability of feature selection was assessed across cross-validation folds, with Jaccard indices ranging from 0.71 to 0.86, indicating robust feature identification despite the limited sample size.

Sensitivity analyses adjusting for age within groups, sex, BMI, eGFR, and chelation status confirmed that the top-ranked biomarkers retained statistical significance after covariate adjustment. Bilirubin glucuronide, 3′-UMP, and vitamin D2 3-glucuronide remained independently associated with WD status in multivariable logistic regression models. Subgroup analysis comparing treatment-naive patients with those receiving chelation therapy revealed consistent metabolic patterns in both strata, although limited sample sizes precluded definitive conclusions regarding treatment effects on the metabolic signature.

As shown in Figure 8, the feature selection process successfully identified minimal metabolite panels that retained high diagnostic accuracy. The different sizes of optimal feature sets between pediatric and adult groups reflect age-specific metabolic complexities in Wilson disease. The consistency of feature importance across multiple algorithms strengthens confidence in the identified biomarkers, supporting their potential clinical utility for age-specific diagnostic applications.

### 3.5 Metabolic pathway analysis

Pathway enrichment analysis was performed to elucidate the biological mechanisms underlying the observed metabolic alterations in Wilson disease. Using the Kyoto Encyclopedia of Genes and Genomes (KEGG) database, differential metabolites were mapped to their corresponding metabolic pathways, revealing distinct patterns of metabolic dysregulation between pediatric and adult patients.

In the pediatric cohort, 23 metabolic pathways were significantly enriched (p<0.05, FDR corrected), with the most prominent alterations observed in pantothenate and CoA biosynthesis (p=0.0012, impact score=0.28), caffeine metabolism (p=0.0018, impact score=0.25), and pyrimidine metabolism (p=0.0023, impact score=0.22). These pathways reflect fundamental disturbances in energy metabolism, nucleotide synthesis, and vitamin processing, consistent with the systemic nature of copper accumulation in developing organisms. Notably, pathways related to amino acid metabolism, including glycine and serine metabolism, methionine metabolism, and glutamate metabolism, were significantly enriched, suggesting disrupted protein homeostasis in pediatric Wilson disease.

The adult cohort demonstrated enrichment in 19 metabolic pathways, with a notable shift towards pathways associated with oxidative stress and neurotransmitter metabolism. Catecholamine biosynthesis (p=0.0008, impact score=0.32), glutathione metabolism (p=0.0015, impact score=0.28), and phenylalanine/tyrosine metabolism (p=0.0021, impact score=0.26) emerged as the most significantly affected pathways. The prominence of the Warburg effect pathway suggests metabolic reprogramming in response to chronic copper toxicity. The catecholamine biosynthesis disruption provides a metabolic correlate for parkinsonian and psychiatric features that predominate in adult WD presentations.

**Figure 9:**
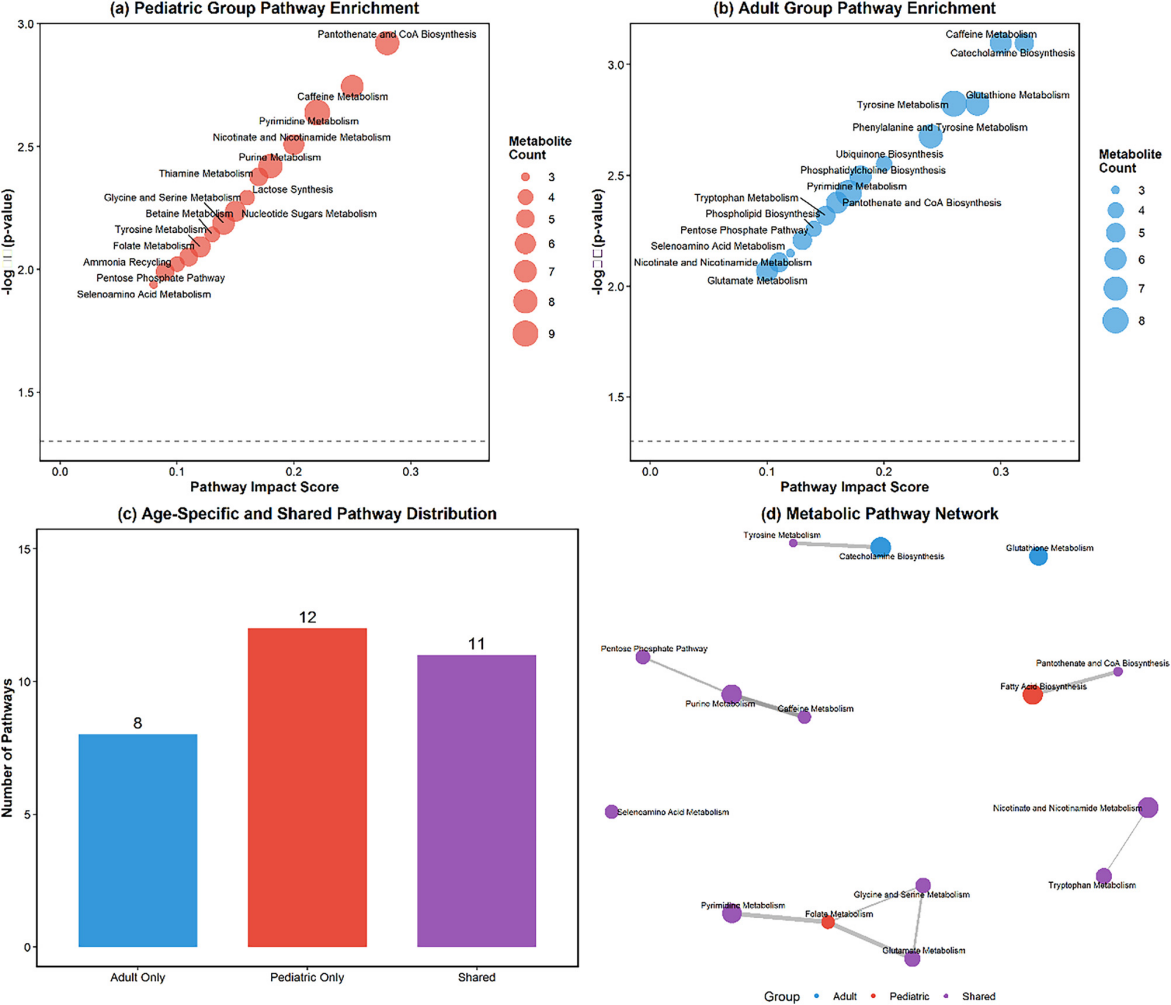
Metabolic pathway analysis revealing age-specific and shared alterations in Wilson disease. (a) Pathway enrichment bubble plot for pediatric group showing 23 significantly affected pathways. (b) Adult group pathway enrichment with 19 altered pathways, notably featuring stress response and neurotransmitter metabolism. (c) Distribution of age-specific and shared pathways demonstrating 11 commonly disrupted pathways. (d) Network visualization illustrating the interconnectedness of metabolic pathways, with node colors representing group specificity and edge width indicating metabolic relationships.

Comparative analysis revealed 11 pathways commonly disrupted across both age groups (Figure 9), representing core metabolic alterations in Wilson disease regardless of age. These shared pathways encompass fundamental processes including nucleotide metabolism (purine and pyrimidine pathways), energy metabolism (pantothenate and CoA biosynthesis, pentose phosphate pathway), and amino acid metabolism (glycine/serine, glutamate, and tryptophan pathways). The consistent disruption of these pathways across age groups suggests they may serve as universal metabolic signatures of Wilson disease, while age-specific pathway alterations likely reflect developmental and disease progression factors.

As shown in Figure 9, the pathway analysis reveals a complex metabolic landscape in Wilson disease, with both age-specific and shared alterations. The prominence of nucleotide metabolism pathways across both groups suggests fundamental disruptions in cellular energy and DNA/RNA synthesis. The age-specific emergence of oxidative stress and neurotransmitter pathways in adults likely reflects the cumulative effects of chronic copper toxicity and may explain the higher prevalence of neurological symptoms in adult patients. These findings provide mechanistic insights into the pathophysiology of Wilson disease and support the development of age-tailored therapeutic interventions targeting specific metabolic vulnerabilities.

## 4 Discussion

This study demonstrates that machine learning can identify urinary metabolic biomarkers capable of distinguishing WD patients from healthy controls with clinically meaningful accuracy, achieving area under the curve values of 0.87 in pediatric and 0.96 in adult cohorts. The findings confirm that metabolic signatures differ substantially between age groups, warranting age-specific diagnostic approaches. This represents the first comprehensive application of ensemble tree-based machine learning methods to age-stratified WD metabolomic diagnosis. XGBoost demonstrated superior performance among the four algorithms evaluated, likely reflecting its advanced regularization techniques and efficient handling of sparse high-dimensional data. These results establish proof-of-concept for age-specific metabolomic diagnostics, though the exploratory nature of these findings necessitates validation in independent cohorts before clinical application.

The superior performance of XGBoost aligns with recent advances in metabolomics-based disease prediction. Chen et al. [16] demonstrated that metabolomic machine learning predictors could achieve exceptional diagnostic accuracy in gastric cancer, with gradient boosting methods showing particular promise. The present findings extend this concept to rare diseases, where XGBoost’s advanced regularization techniques proved particularly valuable for high-dimensional, limited-sample metabolomics data characteristic of WD research. The observed difference in diagnostic performance between age groups merits careful interpretation. Adult patients demonstrated higher classification accuracy compared to pediatric patients, reflected in area under the curve values of 0.96 vs. 0.87. Several factors may contribute to this pattern. Disease duration differed substantially between groups, with adult patients experiencing longer exposure to copper toxicity. Extended disease course may amplify metabolic perturbations, creating more distinct biochemical signatures. The number of differential metabolites identified supports this interpretation, with 109 metabolites altered in adults compared to 68 in pediatric patients. Biological factors may also play a role. Developing organisms possess greater metabolic plasticity and compensatory mechanisms, potentially attenuating biomarker signals in younger patients. Sample size considerations warrant attention as well. The pediatric comparison involved smaller absolute numbers, potentially contributing to wider confidence intervals. These observations suggest that age-specific diagnostic thresholds may optimize clinical performance across developmental stages.

The age-specific metabolic signatures provide mechanistic insights into differential WD presentations across developmental stages. Nucleotide metabolism disruption in pediatric patients reflects heightened vulnerability of rapidly dividing cells to copper-induced oxidative damage. While previous studies have demonstrated that copper toxicity can inhibit ribonucleotide reductase in bacterial systems through metal ion substitution [33], the age-specific predominance of nucleotide pathway alterations in clinical pediatric WD represents a novel observation not previously reported in metabolomic investigations. Copper accumulation may impair enzymes critical for nucleotide biosynthesis, potentially contributing to rapid hepatic decompensation observed in children. Consistent downregulation of pantetheine 4′-phosphate suggests disrupted coenzyme A biosynthesis affecting energy metabolism during growth, another finding not documented in prior WD metabolomic studies. In adults, oxidative stress and neurotransmitter pathway alterations predominate. Chronic copper exposure generates reactive oxygen species through Fenton reactions, overwhelming glutathione-based antioxidant defenses. Catecholamine biosynthesis disruption provides a metabolic correlate for parkinsonian and psychiatric features characteristic of adult WD. Dopamine β-hydroxylase, a copper-dependent enzyme essential for converting dopamine to norepinephrine, is known to be affected by copper dysregulation in Wilson disease [34], 35]. The specific prominence of catecholamine pathway disruption in human adult patients extends previous knowledge and offers novel mechanistic insights linking metabolic alterations to neurological phenotypes.

Several limitations warrant acknowledgment. Subgroup sizes of 10 patients limit statistical power and increase risk of chance findings despite rigorous validation. The study focused on distinguishing WD patients from age-matched controls rather than direct comparison between pediatric and adult patient groups, which would more precisely delineate age-specific vs. disease-related metabolic changes. Additionally, Groups 2 and 3 (adult WD patients with and without advanced disease) were combined for machine learning analysis to maintain adequate sample size for model training and validation. While this approach enabled age-specific biomarker discovery, it precluded independent evaluation of advanced disease patients. Separate analysis of Group 3 alone (n=10) was not feasible given the sample size requirements for reliable machine learning model development with nested cross-validation. Future studies with larger cohorts should evaluate whether metabolic signatures and diagnostic performance differ between early-stage and advanced WD, as disease severity may influence biomarker profiles and model generalizability. Single-center recruitment restricts generalizability across populations with different genetic backgrounds or dietary patterns. Cross-sectional design precludes assessment of metabolic trajectories during disease progression or treatment response. Although sensitivity analyses adjusted for chelation status, residual confounding from medications cannot be fully excluded. Level 2 metabolite identification, while reasonable for discovery, requires confirmation with authentic standards for clinical translation. The absence of an external validation cohort represents the most critical limitation, as all optimization occurred within the same dataset.

The identification of 68 differential metabolites in pediatric patients vs. 109 in adult patients reflects evolving WD pathophysiology across age groups. This contrasts with previous studies that primarily focused on adult populations without age stratification. Qiu et al. [1] first demonstrated distinct metabolomic profiles in WD patients, but did not address age-specific differences or employ machine learning approaches. Similarly, Sarode et al. [2] identified metabolic signatures in WD but focused primarily on adult patients. The present study addresses this critical gap, revealing that diagnostic accuracy improves substantially when age-specific models are employed.

The prominence of nucleotide metabolism pathways in pediatric patients contrasts markedly with oxidative stress and neurotransmitter metabolism pathways in adults. This pattern aligns with clinical observations that hepatic manifestations predominate in children (90 % in the present cohort) while neurological symptoms are more common in adults (60–90 %). The age-dependent directional changes observed in brassicanal C and bilirubin glucuronide suggest that metabolic adaptation mechanisms evolve as disease progresses.

Current diagnostic approaches face significant limitations with serum ceruloplasmin being normal in up to 40 % of WD patients [4]. The metabolomics-based approach offers advantages including non-invasive urine sampling, high diagnostic accuracy exceeding traditional biomarkers, and objective assessment independent of clinical expertise. The LASSO-based feature selection identified parsimonious metabolite panels (7 metabolites for pediatric, three for adult classification) that maintained high diagnostic accuracy, facilitating clinical implementation.

Despite these limitations, the non-invasive urine sampling offers practical advantages over liver biopsy or specialized copper tests. If validated, the compact adult panel of three metabolites could be translated into targeted assays using triple quadrupole mass spectrometry increasingly available in clinical laboratories. Future investigations should prioritize multi-center validation with diverse populations, longitudinal sampling to assess treatment response, and mechanistic studies in experimental models to strengthen biological plausibility.

This exploratory study demonstrates the feasibility of ensemble machine learning for identifying age-specific urinary metabolic signatures in WD. The identification of distinct metabolic signatures between age groups provides valuable insights into WD pathophysiology and supports the development of personalized diagnostic approaches, though independent validation is required before clinical implementation.

## 5 Conclusions

This exploratory study demonstrates the feasibility of ensemble tree-based machine learning methods for identifying age-specific urinary metabolic biomarkers in Wilson disease diagnosis. The integration of UPLC-Q-TOF-MS metabolomics with advanced machine learning algorithms demonstrated exceptional diagnostic performance, with XGBoost achieving AUC values of 0.87 ± 0.03 in pediatric and 0.96 ± 0.02 in adult cohorts through nested cross-validation. The identification of 68 differential metabolites in pediatric patients vs. 109 in adult patients revealed distinct age-related metabolic signatures, with 15 metabolites consistently altered across both age groups representing core Wilson disease pathophysiology.

The metabolic pathway analysis unveiled age-specific disruptions, with nucleotide metabolism pathways predominating in pediatric patients and oxidative stress pathways in adults, providing mechanistic insights into the clinical observation that hepatic manifestations dominate in children while neurological complications become prominent in adults. The LASSO-based feature selection successfully identified parsimonious metabolite panels (7 for pediatric, three for adult classification) that maintained high diagnostic accuracy while facilitating clinical implementation.

Despite the exploratory nature based on a limited single-center cohort, these findings address gaps in Wilson disease diagnosis, offering a non-invasive, objective alternative to current diagnostic challenges where serum ceruloplasmin remains normal in up to 40 % of patients. While encouraging as proof-of-concept, substantial work including multi-center validation and analytical method standardization is required before clinical implementation. Independent validation in geographically diverse cohorts represents the essential next step to determine whether these exploratory biomarkers achieve reproducibility necessary for clinical translation.

## Supplementary Material

Supplementary Material
