# Supplementary material for: Identification of age-specific urinary metabolic biomarkers in Wilson disease using machine learning: a comparative study of ensemble tree models
Source: Open Med (Wars). 2026 Jul 24;21(1):20261415. doi: 10.1515/med-2026-1415 (PMC13392894; doi:10.1515/med-2026-1415)
Supplement: Supplementary file 1 — Supplementary Material [file j_med-2026-1415_suppl_001.docx]

**Supplementary Materials**


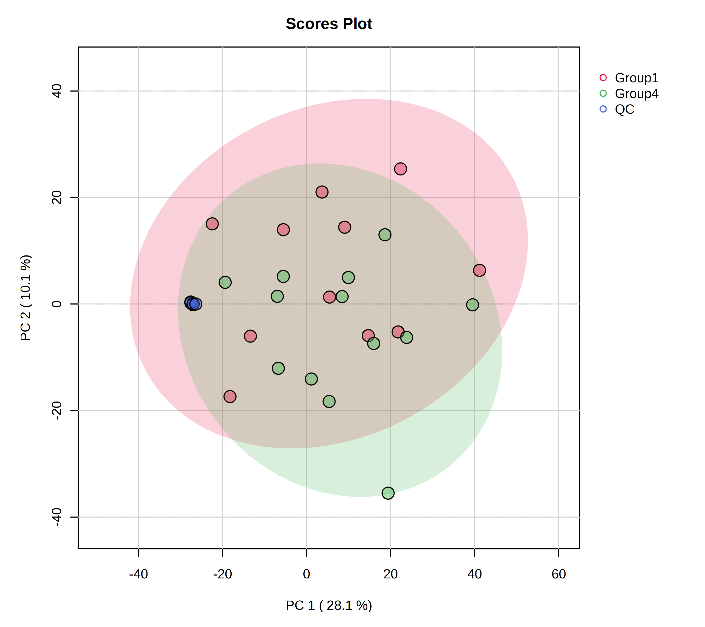

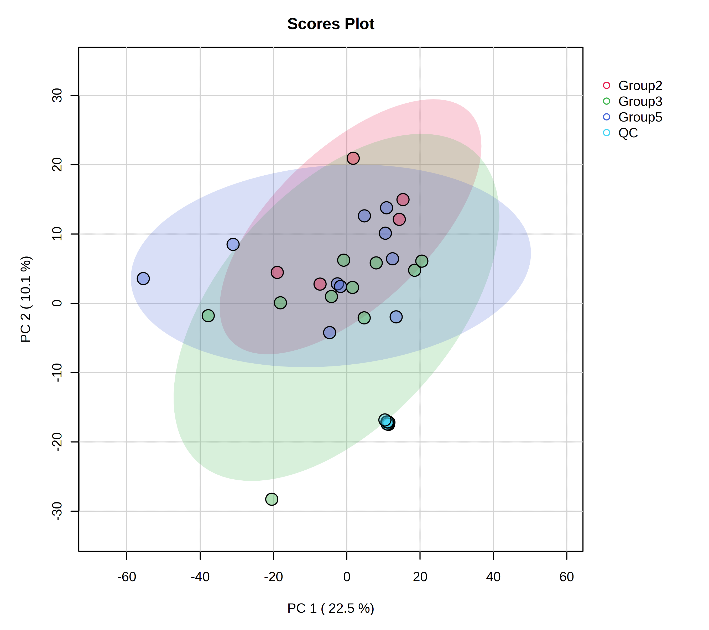


Supplementary Figure S1 Principal component analysis (PCA) score plots demonstrating analytical stability. (a) Pediatric comparison (Group 1 vs Group 4) with QC samples. (b) Adult comparison (Groups 2, 3, and 5) with QC samples. QC samples (quality control, pooled from all samples) cluster tightly together, indicating good instrument stability throughout the analytical sequence.


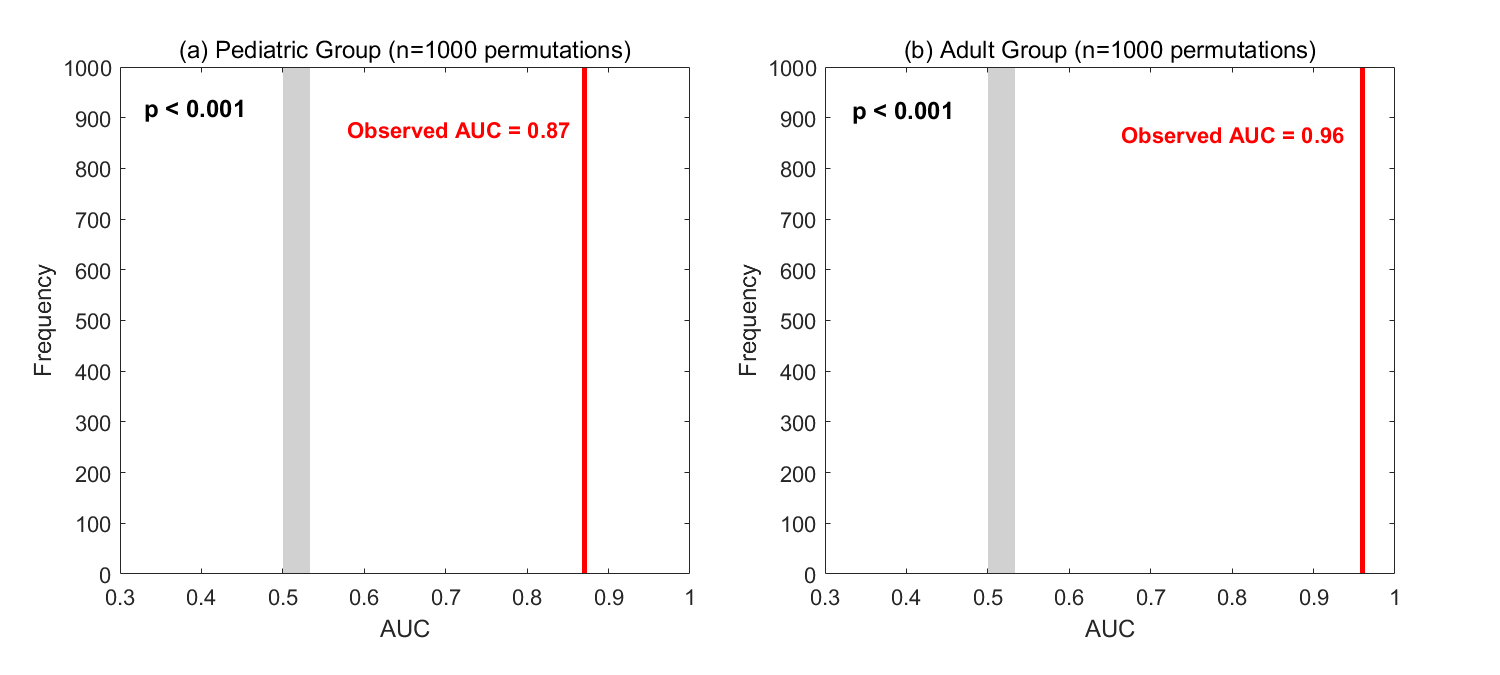


Supplementary Figure S2 Permutation test results validating model performance. Null distributions of AUC values were generated by randomly shuffling class labels 1000 times and retraining the XGBoost model for each permutation. (a) Pediatric group (Group 1 vs Group 4): observed AUC (0.87, red line) significantly exceeded the permutation-based null distribution (p < 0.001). (b) Adult group (Group 2 vs Group 5): observed AUC (0.96, red line) significantly exceeded the null distribution (p < 0.001). Gray histograms represent the distribution of AUC values under the null hypothesis of no association between metabolic profiles and disease status. The concentration of null AUC values around 0.5 confirms that label shuffling eliminates predictive power, while observed AUC values far exceeding chance levels demonstrate genuine classification performance.

Supplementary Figure S3 Learning curves demonstrating model performance as a function of training set size. (a) Pediatric group (Group 1 vs Group 4). (b) Adult group (Group 2 vs Group 5). Blue circles represent training set AUC; red squares represent validation set AUC. Error bars indicate standard deviation from 30 random train-validation splits at each sample size. The pediatric group learning curve shows continued improvement with increasing sample size, suggesting potential benefit from additional samples. The adult group demonstrates a stable performance plateau at larger training sizes, indicating sufficient sample size for reliable classification.

Supplementary Table S1. Commonly altered metabolites identified in both pediatric and adult Wilson disease patients

| No. | Metabolite Name | HMDB ID | Molecular Formula | Ion Mode | Pediatric | Adult |
| --- | --- | --- | --- | --- | --- | --- |
| 1 | 3'-UMP | HMDB0060282 | C₉H₁₃N₂O₉P | ESI+ | Up | Up |
| 2 | Gravelliferone | HMDB0030729 | C₁₉H₂₂O₃ | ESI+ | Up | Up |
| 3 | DHAP(8:0) | HMDB0011685 | C₁₁H₂₁O₇P | ESI+ | Up | Up |
| 4 | Nicotinate D-ribonucleoside | HMDB0006809 | C₁₁H₁₄NO₆⁺ | ESI+ | Up | Up |
| 5 | L-Methionine | HMDB0000696 | C₅H₁₁NO₂S | ESI+ | Up | Up |
| 6 | Brassicanal C | HMDB0038589 | C₁₀H₉NO₃S | ESI+ | Up | Up |
| 7 | Tetrahydroaldosterone-3-glucuronide | HMDB0010357 | C₂₇H₄₀O₁₁ | ESI- | Up | Up |
| 8 | Lithocholate 3-O-glucuronide | HMDB0002513 | C₃₀H₄₈O₉ | ESI- | Up | Up |
| 9 | Pantetheine 4'-phosphate | HMDB0001416 | C₁₁H₂₃N₂O₇PS | ESI- | Up | Up |
| 10 | L-Kynurenine | HMDB0000684 | C₁₀H₁₂N₂O₃ | ESI+ | Down | Down |
| 11 | Methylgallic acid-O-sulphate | HMDB0060005 | C₈H₈O₈S | ESI- | Down | Down |
| 12 | Se-Adenosylselenohomocysteine | HMDB0011117 | C₁₄H₂₀N₆O₅Se | ESI- | Down | Down |
| 13 | 7-Methylxanthine | HMDB0001991 | C₆H₆N₄O₂ | ESI- | Down | Down |
| 14 | Bilirubin glucuronide | HMDB0010332 | C₃₉H₄₄N₄O₁₂ | ESI+ | Down | Up |
| 15 | (2E)-Hexacosenoyl-CoA | HMDB0062228 | C₄₇H₈₄N₇O₁₇P₃S | ESI+ | Down | Up |

*Note: Up, upregulated in WD patients compared with healthy controls; Down, downregulated. Metabolites 1-13 showed concordant changes in both age groups; metabolites 14-15 showed age-dependent directional changes. HMDB, Human Metabolome Database; ESI+/ESI-, positive/negative electrospray ionization mode.*

Supplementary Table S2. Representative differential metabolites in Wilson disease patients

(a) Pediatric group (Group 1 vs Group 4, total n=68)

| No. | Metabolite Name | HMDB ID | Molecular Formula | Ion Mode | Regulation |
| --- | --- | --- | --- | --- | --- |
| 1 | Vanillactic acid | HMDB0000913 | C₁₀H₁₂O₅ | ESI+ | Up |
| 2 | 3'-UMP | HMDB0060282 | C₉H₁₃N₂O₉P | ESI+ | Up |
| 3 | Gravelliferone | HMDB0030729 | C₁₉H₂₂O₃ | ESI+ | Up |
| 4 | DHAP(8:0) | HMDB0011685 | C₁₁H₂₁O₇P | ESI+ | Up |
| 5 | CMP-N-acetylneuraminic acid | HMDB0001176 | C₂₀H₃₁N₄O₁₆P | ESI+ | Up |
| 6 | 4-Pyridoxic acid | HMDB0000017 | C₈H₉NO₄ | ESI+ | Up |
| 7 | Hippuric acid | HMDB0000714 | C₉H₉NO₃ | ESI+ | Up |
| 8 | Nicotinate D-ribonucleoside | HMDB0006809 | C₁₁H₁₄NO₆⁺ | ESI+ | Up |
| 9 | Tetrahydrofolic acid | HMDB0001846 | C₁₉H₂₃N₇O₆ | ESI+ | Up |
| 10 | 5-Methylquinoxaline | HMDB0033178 | C₉H₈N₂ | ESI+ | Down |
| 11 | 20-Oxo-leukotriene E4 | HMDB0012642 | C₂₃H₃₅NO₆S | ESI+ | Down |
| 12 | Estradiol | HMDB0000151 | C₁₈H₂₄O₂ | ESI+ | Up |
| 13 | 3,7-Dimethyluric acid | HMDB0001982 | C₇H₈N₄O₃ | ESI+ | Down |
| 14 | 3-Oxodecanoic acid | HMDB0010724 | C₁₀H₁₈O₃ | ESI+ | Up |
| 15 | Bilirubin glucuronide | HMDB0010332 | C₃₉H₄₄N₄O₁₂ | ESI+ | Down |

(b) Adult group (Group 2 vs Group 5, total n=109)

| No. | Metabolite Name | HMDB ID | Molecular Formula | Ion Mode | Regulation |
| --- | --- | --- | --- | --- | --- |
| 1 | 3'-UMP | HMDB0060282 | C₉H₁₃N₂O₉P | ESI+ | Up |
| 2 | Ethosuximide | HMDB0014731 | C₇H₁₁NO₂ | ESI+ | Up |
| 3 | Gravelliferone | HMDB0030729 | C₁₉H₂₂O₃ | ESI+ | Up |
| 4 | DHAP(8:0) | HMDB0011685 | C₁₁H₂₁O₇P | ESI+ | Up |
| 5 | Nicotinate D-ribonucleoside | HMDB0006809 | C₁₁H₁₄NO₆⁺ | ESI+ | Up |
| 6 | 5-Acetylamino-6-formylamino-3-methyluracil | HMDB0011105 | C₈H₁₀N₄O₄ | ESI+ | Up |
| 7 | Deoxyuridine | HMDB0000012 | C₉H₁₂N₂O₅ | ESI+ | Down |
| 8 | Yayoisaponin C | HMDB0033401 | C₅₁H₈₄O₂₅ | ESI+ | Up |
| 9 | N(omega)-Hydroxyarginine | HMDB0004224 | C₆H₁₄N₄O₃ | ESI+ | Up |
| 10 | Hordatine B glucoside | HMDB0030460 | C₃₅H₅₀N₈O₁₀ | ESI+ | Up |
| 11 | Campesteryl brassidate | HMDB0036290 | C₅₀H₈₈O₂ | ESI+ | Up |
| 12 | 4-Hydroxybenzoic acid | HMDB0000500 | C₇H₆O₃ | ESI+ | Down |
| 13 | Imidazolepropionic acid | HMDB0002271 | C₆H₈N₂O₂ | ESI+ | Down |
| 14 | Brassicanal C | HMDB0038589 | C₁₀H₉NO₃S | ESI+ | Up |
| 15 | L-Methionine | HMDB0000696 | C₅H₁₁NO₂S | ESI+ | Up |

*Note: Representative differential metabolites selected from the complete list (68 metabolites in pediatric group, 109 in adult group). Complete metabolite lists are available in the supplementary Excel file. Metabolites were identified based on VIP > 1, fold change > 2, and p < 0.05 (FDR corrected). Up, upregulated in WD patients; Down, downregulated. HMDB, Human Metabolome Database; ESI+/ESI-, positive/negative electrospray ionization mode.*
